# A Treatise on Diphtheria

**Published:** 1887-11

**Authors:** 


**A Treatise on Diphtheria, Historically and Practically Considered; including Croup, Tracheotomy and Intubation.** By Dr. A. Samie, Docteur eu Médicine Ancien des Hôpitaux de Paris, Membre de la Société Anatomique, etc. Translated, annotated and the surgical anatomy added; illustrated with a full-page colored lithograph and many wood engravings, by Henry Z. Gill, A. M., M. D., LL.D., late Professor of Operative and Clinical Surgery in the Medical Department of the University of Wooster, at Cleveland, O., Member of the American Medical Association, etc. St. Louis, Mo.: J. H. Chambers & Co. 1887.

This book has attracted great attention from the profession, "taking," as it does, "every feature of the subject under consideration, tracing its history down through the centuries, the clinical observations, the pathological manifestations; microscopical and clinical examination and its inoculation." The translation is excellent, as those who have the work in the original can testify. The anatomical addition is in keeping with the rest of the work. As one reads, he becomes infatuated with the work, and many of the points which were not made clear by the writings of Bretonneau, Trouseau, and other writers, are settled here. It is not too voluminous for any practitioner, and every word seems necessary. The book is excellently illustrated with forty-one wood cuts.

